# Spatially resolved fetal and maternal cell contributions to severe preeclampsia across gestation

**DOI:** 10.1126/sciadv.aed8964

**Published:** 2026-07-24

**Authors:** Yara E. Sanchez-Corrales, Theodoros Xenakis, Jose J. Moreno-Villena, Leysa Forrest, Neil J. Sebire, Elizabeth C. Rosser, Lucy R. Wedderburn, Sergi Castellano, Sara L. Hillman

**Affiliations:** ^1^ Genetics and Genomic Medicine Research & Teaching Department, UCL Great Ormond Street Institute of Child Health, London, UK.; ^2^ UCL Genomics, UCL Great Ormond Street Institute of Child Health, London, UK.; ^3^ Department of Histopathology, Great Ormond Street Hospital for Children NHS Foundation Trust, London, UK.; ^4^ Centre for Adolescent Rheumatology Versus Arthritis at UCL, UCL Hospitals and GOSH, London, UK.; ^5^ Department of Ageing, Rheumatology and Regenerative Medicine, UCL Division of Medicine, UCL, London, UK.; ^6^ Infection, Immunity and Inflammation Research and Teaching Department, UCL Great Ormond Street Institute of Child Health, London, UK.; ^7^ NIHR Biomedical Research Centre at Great Ormond Street Hospital NHS Foundation Trust, London, UK.; ^8^ University College London Hospitals (UCLH) NHS Foundation Trust, London, UK.; ^9^ Institute for Women's Health, University College London (UCL), London, UK.

## Abstract

The molecular and cellular pathophysiology of preeclampsia remains poorly understood, but it is increasingly clear that in addition to the placenta, other tissues of the fetal-maternal interface advance the disease. Here, we distinguish fetal and maternal contributions to the early and late presentation of severe preeclampsia by interrogating, across time and space, tissues and cell types relevant to the disease. Accounting for gestational age in a third trimester preterm cohort, we find single-cell and spatial molecular signatures of concerted hypoxia, angiogenic imbalance, fibrosis, and aberrant metabolism in the placenta. In addition, we report maternal immune signatures such as mitochondrial dysfunction and interferon signaling extending to the myometrium and chorioamniotic membranes, likely contributing to the systemic inflammation and endothelial dysfunction in the mother, with impaired fetal cell interactions with endothelial cells in the myometrium contributing to it. These tissue- and cell-specific responses are potential targets for therapy, with their prompt consideration in early gestational disease likely beneficial because of its aggravated molecular presentation. Thus, timely intervention during gestation could change the extremely poor prognosis of severe preeclampsia.

## Teaser

Fetal and maternal contributions to severe preeclampsia unraveled in space and time with single-cell and spatial transcriptomics.

## INTRODUCTION

The fetal maternal interface (FMI) is the site of interaction between specialized fetal cells (trophoblasts) and maternal cells during pregnancy (*1*). It includes the placental villi, where trophoblasts are in contact with maternal blood, and extends to the adjacent decidua basalis (at the site of placentation) and decidua parietalis (surrounding the fetus), where trophoblasts contact maternal cells from the modified myometrium. These interactions remain poorly defined, but they are essential to understand, prevent, and treat pregnancy complications such as preeclampsia (PE).

PE is recognized as a placental pathology (*2*) with systemic consequences, including new onset maternal hypertension and proteinuria at or after 20 weeks of gestation (*3*). It is a leading cause of maternal and fetal morbidity and mortality, affecting 2 to 4% of pregnancies globally with more than 46,000 maternal and 500,000 fetal and neonatal deaths per year (*4*). Early in pregnancy, the placental cytotrophoblast (CTB) cells differentiate into syncytiotrophoblasts (STBs), which contact maternal blood, or extravillous trophoblasts (EVTs), which invade through the decidua and remodel the spiral arteries of the myometrium (*5*). PE is associated with a shallow EVT invasion and defective spiral artery remodeling, leading to placental stress and ischemia, as well as systemic maternal endothelial dysfunction and endovascular inflammation (*3*, *6*). PE is a highly heterogeneous disease for which no effective treatment exists (*3*).

Recent studies have applied single-cell transcriptomics to placental villous tissue from pregnancies complicated by PE and characterized important transcriptional changes in trophoblast and maternal cells (*7*–*12*). In PE, EVTs have gene expression changes related to migration and cell death (*8*, *9*), whereas STBs have changes related to angiogenic imbalance (*7*) and senescence (*10*). Among maternal cells affected in PE are endothelial cells and immune cells, such as macrophages and T cells (*7*). Moreover, Admati *et al.* (*7*) quantified differences between early PE (≤34 gestational weeks) and late PE (>34 gestational weeks), confirming the heterogeneity of the disease. In addition to placental dysfunction, recent evidence suggests aberrant transcription in other locations of the FMI such as the decidua basalis (*10*), endometrial tissue (*11*), and chorioamniotic membranes (CAMs) (*13*). These studies often interrogate one tissue, missing the spatial heterogeneity of cell interactions within the FMI that underlies disease presentation and could reveal novel therapeutic targets.

Here, we focus on the severe presentation of the disease, which often involves maternal multiorgan dysfunction (*14*) and preterm birth (<37 gestational weeks), with potential long-term health complications for both mother and fetus (*3*). Despite the clinical and molecular importance of the placenta, elucidation of PE in its most florid state in the wider FMI is lacking. We present a spatially resolved, multitissue single-cell transcriptomic analysis of the entire FMI in a carefully phenotyped cohort of patients with severe PE and gestational matched controls, spanning 25 to 37 gestational weeks. Correcting for gestational age, we quantify both suspected and novel fetal and maternal contributions to disease, per tissue and cell type, identifying distinct transcription underlying the early extreme and late moderate dysfunction in severe PE. In addition to concerted hypoxia, angiogenic imbalance, fibrosis, and aberrant metabolism in the placenta, we reveal previously unknown maternal immune signatures, including changes in macrophages, mitochondrial dysfunction, and interferon (IFN) signaling across the FMI. We also confirm and quantify the dearth of EVT invasion into the myometrium in severe PE, found in conjunction with impaired and reduced presence of eEVTs around endothelial cells. Together, we offer an explanation to systemic inflammation and endothelial dysfunction in the mother, as well as potential targets for therapy.

## RESULTS

### Study cohort and multitissue sampling across the FMI

**Fig. 1.**
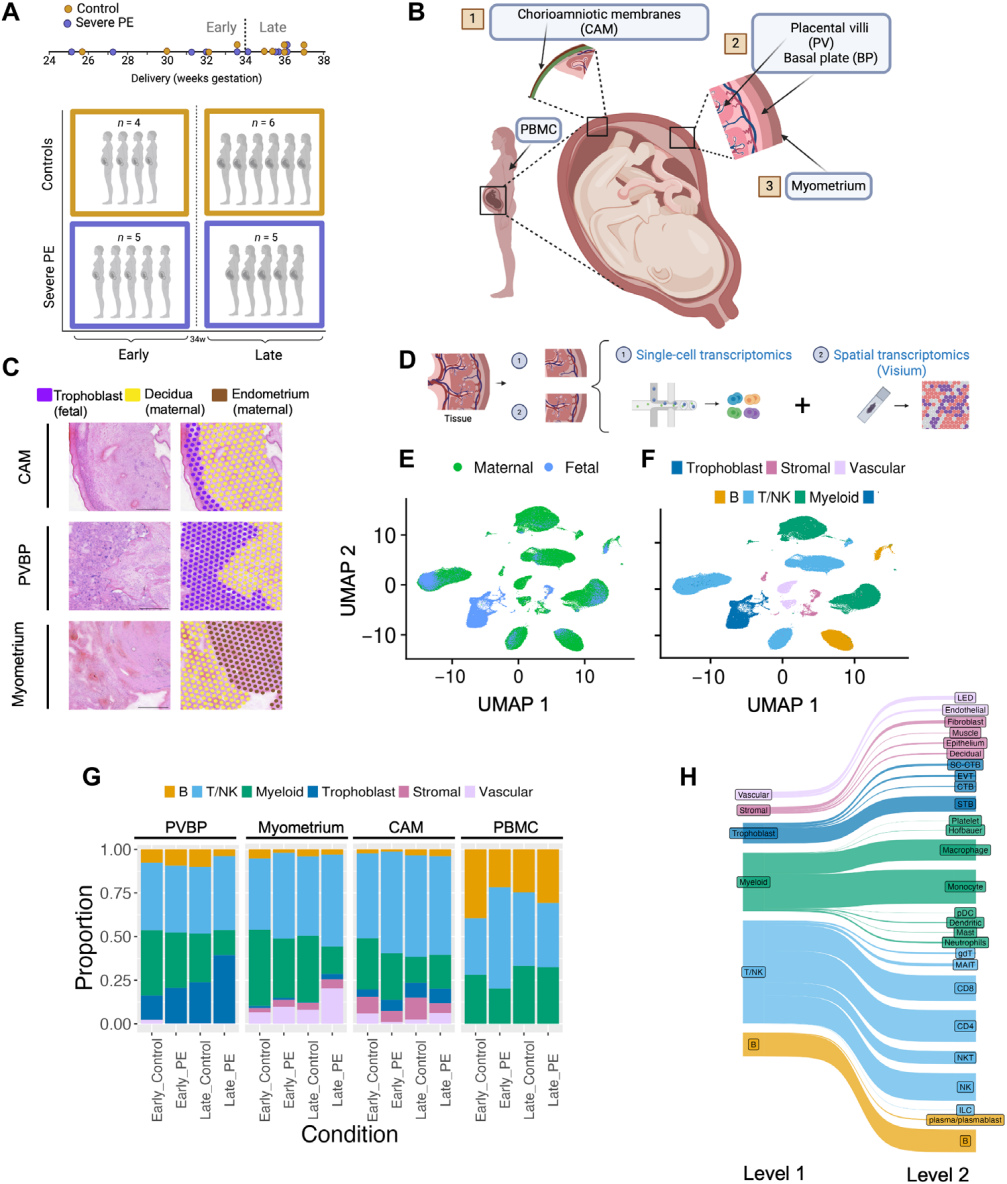
Multitissue single-cell atlas of the human FMI with spatial context in severe PE. (**A**) Cohort of 20 donors to study severe PE (*n* = 10 cases and *n* = 10 controls) with gestational ages from 25 to 37 weeks (w) across early (≤34 weeks) and late disease presentation (>34 weeks). (**B**) Multitissue sampling per donor, including maternal blood (PBMCs), CAM, PVBP, and myometrium. (**C**) Fetal and maternal population sampling across the entirety of the FMI. Scale bars, 500 μm. (**D**) Single-cell transcriptomics and Visium spatial transcriptomics run on the same sample for each donor to spatially resolve the effects of severe PE across the FMI. (**E**) Uniform manifold approximation and projection (UMAP) of fetal and maternal cells, identified using their genetic variation (SNPs) and trophoblasts markers. (**F**) UMAP with the six major cell groups annotated (level 1 annotation), including immune cell groups (B cell, T/NK cell, and myeloid) and nonimmune cell groups (trophoblast, stromal, and vascular). (**G**) These six groups were present across conditions. (**H**) Clustering within each group from the level 1 annotation resulted in annotations with increasing granularity, with 27 cell types in the level 2 annotation. Panels (A), (B), and (D) were created in BioRender. S. Castellano (2026), https://biorender.com/mmkirrp, https://BioRender.com/nmhsibd, and https://BioRender.com/5oeouoo, respectively.

To study severe PE at a single-cell resolution across the entirety of the human FMI, we recruited a cohort of 20 donors, of whom 10 were experiencing severe PE from 25 to 37 weeks of gestational age, alongside 10 gestationally matched (±1 week) controls, hence spanning across early (≤34 weeks) and late (>34 weeks) disease (Fig. 1A and Table 1). As pregnancy involves dynamic molecular changes from week to week, a comparison including gestational-age matched controls is more meaningful than comparing preterm cases to term healthy controls. Gestation matched controls were from iatrogenic preterm deliveries that did not experience PE or any other maternal chronic disease (Materials and Methods and cohort description in Table 1). Donors were tested for the presence of infection by standard clinical care but also with metagenomics (Supplementary Text and figs. S1 and S2). As a result, one control donor was excluded from our comparative analysis because of detected infection (Supplementary Text and figs. S1 and S2).

**Table 1.** Severe PE cohort description. BMI, body mass index; BP, blood pressure; uPCR, urine protein-creatinine ratio; PE, severe preeclampsia; HELLP, hemolysis, elevated liver enzymes, and low platelets; AKI, acute kidney injury; IUGR, intrauterine growth restriction; UmA, umbilical artery; EDF, end diastolic flow; MVM, maternal vascular malperfusion.

ID	Gestation of disease onset (weeks)	Gestation of delivery (weeks)	Steroids < 1-week birth	Maternal condition	Maternal age	Ancestry	BMI	Parity	Highest BP (mmHg)	Highest uPCR (mg/mmol)	Blood abnormalities	Birth weight (g)	Sex of baby	Fetal condition	Abnormal fetal dopplers	Histopathological abnormality
FVQ		25 + 5	Y		43	South American	32	0				1122	M			Nil
FLR		30 + 1	Y		36	White British	19	1				1469	F			Acute chorioamnionitis
FJJ		30 + 3	Y		31	Southeast Asian	29	0				1337	M			Acute chorioamnionitis
FVB		32 + 1	Y		41	Southeast Asian	23	0				2110	M			Nil
FCM	24 + 6	25 + 1	Y	PE	33	White British	19	0	173/123	212	Transaminitis	552	F	IUGR 2nd centile	Reversed UmA EDF	MVM
FAM	26 + 3	27 + 2	Y	PE	40	Black African	29	2	202/116	449	Transaminitis AKI	715	F	IUGR <5th centile	UmbA PI >95th centile	MVM
FGS	30 + 4	31 + 2	Y	PE	40	White British	32	0	190/109	1101	AKI	1500	M	IUGR <5th centile		MVM
FEP	28 + 6	32 + 0	Y	PE	40	Middle Eastern	22	0	168/100	203	Transaminitis	1238	M	IUGR 2nd centile	UmbAPI>95th centile	MVM
FVS	29 + 2	33 + 4	Y	PE	41	White British	25	0	168/92	630	Transaminitis AKI	1460	M	IUGR <1st centile		MVM
FLJ		33 + 4	Y		29	White British	20	0				2207	F			Nil
FJD		35 + 2	N		43	South Asian	25	0				2430	F			Nil
FVT		36 + 0	N		39	Southeast Asian	24	1				2820	M			Nil
FYI		36 + 2	Y		35	White British	22	0				2840	M			
FNS		37 + 0	N		35	South Asian	37	1				2255	F			
FRK		37 + 0	N		25	White (other)	27	1				2800	M			Nil
FFM	33 + 4	34 + 1	Y	PE	37	White British	22	0	191/94	591	Transaminitis	1530	F	IUGR <1st centile	Absent UmA EDF	MVM
FRH	34 + 4	35 + 4	Y	PE	31	Indian Asian	22	0	189/98	1116	Transaminitis	2430	M			MVM
FSH	35 + 3	35 + 5	Y	PE+ HELLP	39	White British	20	0	146/101	59	Transaminitis AKI	2256	M			Accelerated villous maturation
FND	35 + 6	36 + 1	Y	PE	33	Northern European	22	0	170/104	43	Transaminitis	2500	M	IUGR 1st centile	Low CPR.	Focally accelerated villous maturation
FMM	34 + 2	36 + 1	Y	PE	32	White British	20	0	160/93	360		2260	M			Nil

We designed a systematic sampling strategy to profile cell populations from multiple tissues of the FMI for each donor. We sampled the placental villi and basal plate (PVBP), the CAMs, and myometrium (Fig. 1, B and C). This ensured the inclusion of fetal and maternal populations from the entirety of the FMI: placental villi (fetal), complete decidua basalis (maternal, from the basal plate and myometrium), chorion (fetal), and decidua parietalis (maternal, from the uterus). We also sampled maternal peripheral blood mononuclear cells (PBMCs) at delivery per donor to study peripheral molecular signatures associated to disease. Careful sampling of tissues across cases and controls at a single-cell resolution ensured the power to identify suspected and novel signatures of the disease across gestation (Materials and Methods and Supplementary Text)

### Constructing a multitissue single-cell atlas of the FMI

To assess cell populations across the FMI, we performed single-cell transcriptomics on the FMI tissues and PBMCs without cell type enrichment (Fig. 1D). We analyzed 292,773 high-quality cells (fig. S3). To classify cells as fetal or maternal, we leveraged their genetic variation (*15*) as well as gene expression in fetal trophoblasts (Fig. 1E and figs. S5 and S6). For donors and tissues, we manually annotated clusters on the basis of differential gene expression and known markers (figs. S7 to S12). We first identified six major cell groups (Fig. 1F, level 1 annotation), including immune cell groups [B cell, T/natural killer (NK) cell, and myeloid] and nonimmune cell groups (trophoblast, stromal, and vascular) (Fig. 1G). We repeated the clustering in each group (Fig. 1H), which resulted in 27 cell types (level 2 annotation) and 59 cell types (level 3 annotation) (markers per tissue and cell type in figs. S7 to S12). Using single-cell RNA sequencing, we recovered both fetal trophoblasts (STBs, CTBs, and EVTs) and maternal immune cells (Supplementary Text and table S3) (*16*, *17*). To complement this reference annotation and provide spatial context to it, we performed Visium spatial transcriptomics (10x Genomics) on adjacent cuts from the same tissue sample (Fig. 1, B to D, and fig. S4). We used this FMI single-cell atlas to inform the spatially resolved quantification of maternal and fetal cell contributions to severe PE.

### Importance of correcting for gestational age

**Fig. 2.**
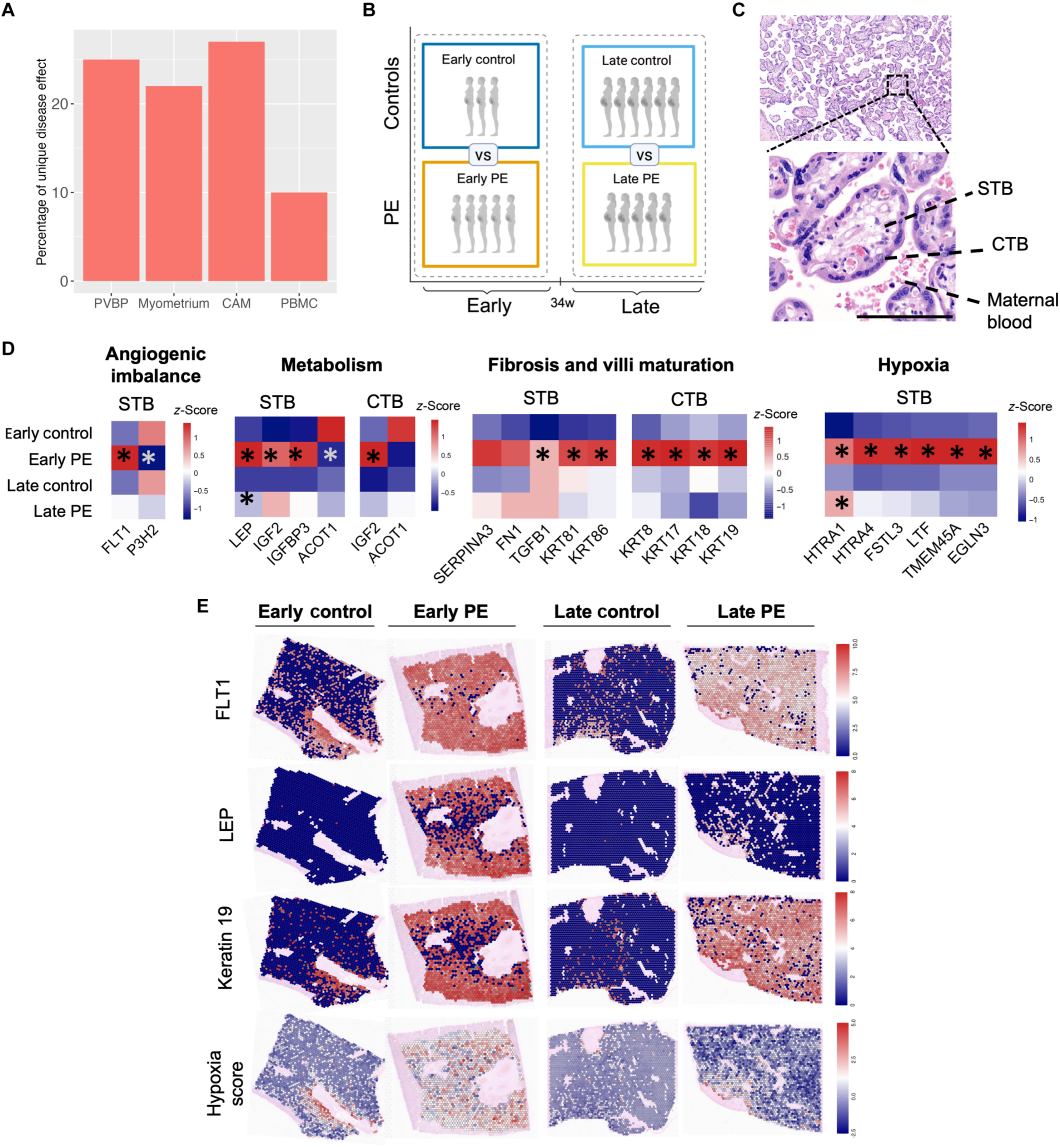
Severe PE involves angiogenesis imbalance, metabolic alterations, and hypoxia of the placental villi. (**A**) Unique contribution of severe PE to the molecular signatures per tissue (in %). (**B**) Case-control experimental design considering gestational age and PE classification into early and late severe disease with a GLM. Created in BioRender. S. Castellano (2026), https://biorender.com/pqxmdg5. (**C**) Hematoxylin and eosin (H&E) staining of the placenta villi with CTBs and STBs. Scale bar, 100 μm. (**D**) Heatmaps of gene expression differences from our GLM. Expression is normalized and scaled in a *z*-score across conditions per gene. *P < 0.05. (**E**) Spatial gene expression in the placenta villi in representative samples per condition. Spatial plots with log-normalized counts. H&E staining of these tissues is also shown in fig. S17.

We separately quantified the contribution of both gestational age and severe PE to molecular signatures of disease. We used the differential abundance of cell types as a proxy for this contribution, with unique abundance changes from gestational age (late versus early) and disease (severe PE versus control) revealing the impact of gestation and disease, respectively. The unique contribution of FMI tissues to severe PE is around 25%, whereas it is only 10% in PBMCs (Fig. 2A and fig. S13). This agrees with severe PE being a placental disease, with the addition that cell contributions in the CAMs and myometrium are similar to those from the PVBP. A relevant but more modest contribution comes from peripheral tissues, as evidenced in PBMCs (Fig. 2A). Most molecular differences thus are affected or confounded by gestation, even when cases and controls are matched within a week, underscoring taking gestational age into account in prenatal disease.

We reached a comparable conclusion using gene expression as a proxy for the contribution of each cell type to gestation and disease. This is achieved with a generalized linear model (GLM), whose parameterization quantifies early and late disease as well as the average disease effect and average gestational age effect (figs. S13 and S14 and Supplementary Text). This model identifies those cell types that contribute the most to disease in severe PE, early and late (Fig. 2B and fig. S14), which we discuss per tissue.

### Placental villi have spatially concerted pathological gene expression

We first examined the placental villi, fetal structures where gases and nutrients are exchanged between maternal and fetal blood, formed by the multinucleated STBs and their precursors CTBs (Fig. 2C). We identified mature STBs (*n* = 20,734) and CTBs (*n* = 962) (table S3) as well as smaller clusters of CTBs proliferating (*n* = 323) and STB precursors (*n* = 52), which express *syncytin-2* (*ERVRD-1*), implicated in trophoblast fusion (*18*). We found that STBs and CTBs contribute the most to early disease (fig. S15D). STBs in early PE have significantly higher expression (*P* > 0.05) of *Fms-related receptor tyrosine kinase 1* (*FLT1*), known to be clinically related to angiogenic imbalance (*3*). Conversely, *prolyl 3-hydroxylase 2* (*P3H2*), whose activity is angiogenic, is significantly down-regulated in early PE (Fig. 2D and figs. S15E and S16A) (*19*). We also found overexpression of genes altering the metabolism. *Leptin* (*LEP*) was significantly up-regulated in early and late disease, consistent with previous reports (Fig. 2D and fig. S16B) (*20*, *21*). We also confirmed significant up-regulation of insulin-like growth factor-binding protein 3 (*IGFBP3*) in STBs and *insulin-like growth factor 2* (*IGF2*) in both STBs and CTBs (*22*), alongside down-regulation of *acyl-coenzyme A thioesterase 1* (*ACOT1*), implicated in lipid metabolism (Fig. 2D) (*23*).

Furthermore, we found dysregulation of multiple keratins, mainly in early disease. These include significant up-regulation of *KRT18*, *KRT8*, and *KRT19* in CTBs (Fig. 2D and fig. S16D, *P* > 0.05), which is associated with PE and implicated in trophoblast differentiation (*24*). We find up-regulation of additional keratins such as *KTR17* in CTBs and *KRT81* and *KRT86* in STBs (Fig. 2D). Keratin dysregulation has been associated with fibrosis in other tissues (*25*). In addition, other genes promoting fibrosis and extracellular matrix deposition, such as *Transforming Growth Factor beta 1* (*TGFB1*), *fibronectin-1* (*FN1*) (*26*), and *SERPINA3* (*27*), in STBs are overexpressed (Fig. 2D), suggesting that placental fibrosis broadly contributes to severe PE.

Last, we identified up-regulated genes in STBs, particularly in early PE, related to hypoxia. These include *high temperature-requirement A4* (*HTRA4*) and *HTRA1* (*28*), *Follistatin-like 3* (*FSTL3*) (*29*), *Transmembrane protein 45A* (*TMEM45A*) (*29*), and *Egl-9 Family hypoxia Inducible Factor 3* (*EGLN3*) (Fig. 2D and fig. S16) (*30*). We also found an STB subpopulation, overexpressing these hypoxic genes, that is unusually abundant in early and late disease (fig. S15, B and C). They may underlie the ischemia and pathological increase of hypoxia in PE (*31*).

We next investigated the spatial expression of these genes in the villi from the PVBP, excluding vasculature and decidua (figs. S17 and S18). It agrees with our single-cell differential expression results on STBs and CTBs (Fig. 2). In addition, we quantified the spatial variation of genes overexpressed in severe PE and found homogeneous gene expression across the villous region. This supports a concerted and shared stress response of trophoblasts from the villous tree (Fig. 2E).

Together, our results confirm the central role of placental villi formed by STBs and CTBs in disease presentation (*2*), reassuring the robust sampling of relevant cell types in our cohort and power to detect differences in our case-control comparison. Moreover, we identify a homogeneous spatial response of the villi in early and late disease, accentuated however in its early presentation. We next investigated nonvillous trophoblast populations from other FMI tissues.

### EVTs in severe PE change in abundance at the decidua basalis

**Fig. 3.**
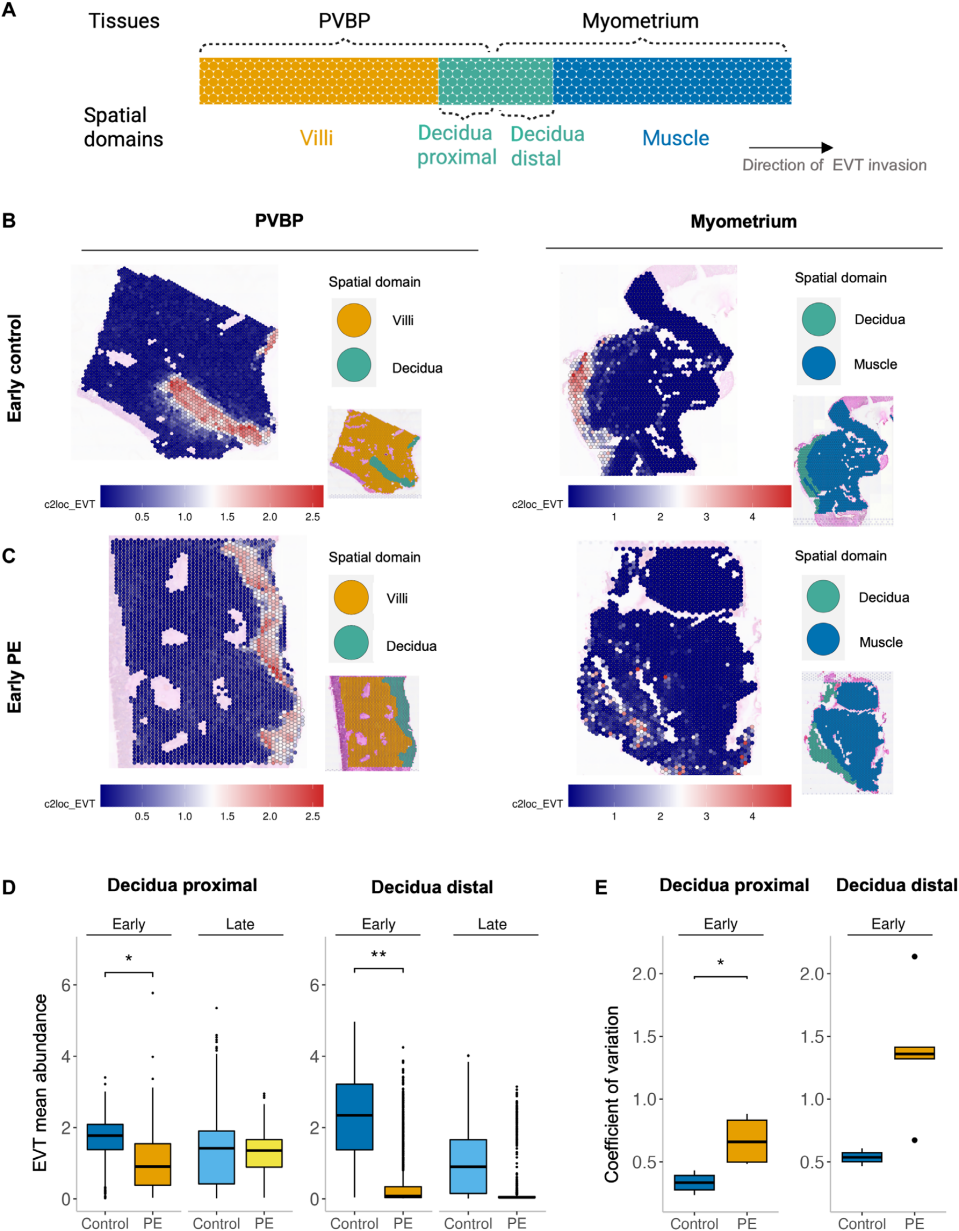
EVT spatial depletion in severe PE. (**A**) Schematic of the direction of EVT invasion across tissues and spatial domains at the placenta bed. The PVBP includes the spatial domains villi and the adjacent decidua proximal to it. The adjacent myometrium continues with the decidua basalis, distal to the villus, and the muscle. Created in BioRender. S. Castellano (2026), https://biorender.com/zj3w194. (**B**) Deconvoluted abundance of EVTs at the decidua basalis and myometrium in a representative early control. (**C**) Deconvoluted abundance of EVTs at the PVBP and myometrium in a representative early severe PE. H&E staining of these tissues is shown in fig. S19. (**D**) The spatial abundance of EVTs in the decidua proximal and distal to the villus is reduced in early PE compared to early controls (*P < 0.05 and **P < 0.01, mixed-effects model). (**E**) Coefficient of variation of spatial EVT abundance across the decidua basalis, proximal and distal to the villi in the PVBP, with significant variation in early PE (*P* < 0.01) (Wilcoxon rank-sum exact test).

Although defective spiral artery remodeling by the fetal EVT has been implicated in PE (*2*, *3*, *6*), it is unclear whether this is due to decreased density or depth in the invasion of EVTs, with immunohistochemistry providing contradictory interpretations (*32*, *33*). Our experimental strategy ensured the sampling of the full decidua basalis into two spatially adjacent cuts (Fig. 3A). This allowed us to evaluate EVT spatial distribution. We did this by defining spatial domains as anatomical regions in a tissue, sharing histology and gene expression with a specific cell composition. In the PVBP, we distinguished two domains: the placental villi (fetal origin) and the decidua basalis (maternal origin) next to the villi (decidua proximal) (Fig. 3A and fig. S19). Similarly, we separated the myometrium into two spatial domains: the decidua distal to the villi (a continuation of the decidua basalis neighboring the muscle) and muscle (Fig. 3A and fig. S19), both of maternal origin.

We detected a significant decrease of EVTs in early PE in the decidua proximal to the villi (*P* < 0.05) (Fig. 3, B to D), which increased with distance to the villi (decidua proximal, *P* < 0.01) (Fig. 3D), pointing to a decreased depth of invasion. We also quantified the spatial distribution of these EVTs and found that they are sparsely distributed across the decidua basalis (Fig. 3E). This is however less evident in late disease. Some EVTs are present in the muscle in the myometrium, but their numbers do not allow for statistical interpretation (fig. S20D). This is, however, confirmed by in situ spatial transcriptomics at a single-cell resolution (Xenium) on the myometrium tissue of a subset of early donors (four cases and two controls) (Materials and Methods and fig. S20E). Thus, failure of spiral artery remodeling by EVTs in severe PE is from both reduced density and depth of invasion, explaining the apparent contradiction of a previous study (*32*).

### Neighborhoods of eEVTs and maternal vasculature decline in early PE

**Fig. 4.**
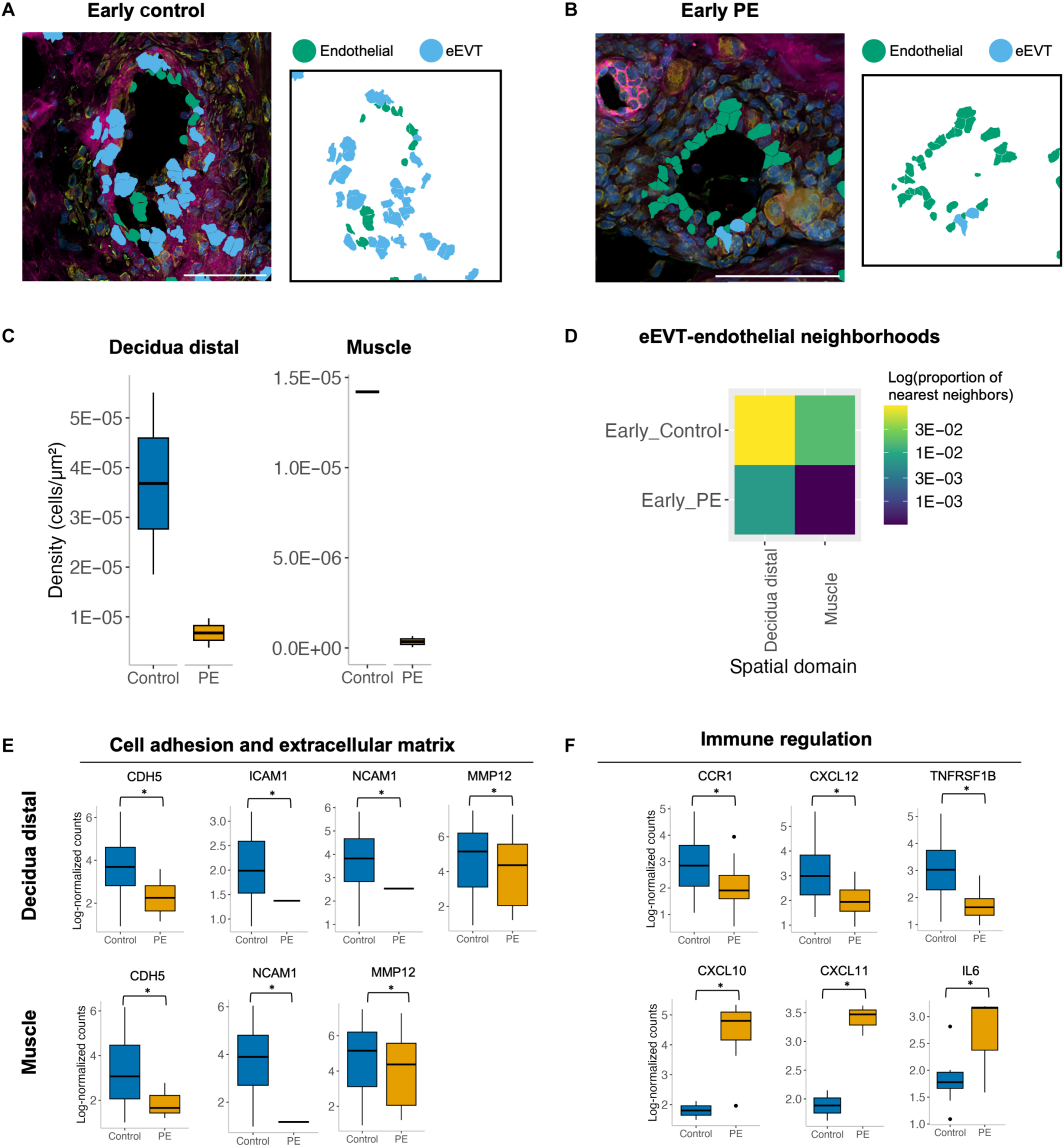
eEVTs are less frequently associated with endothelial cells in early severe PE. (**A** and **B**) Representative early control (A) and early PE (B) vessels from the myometrium profiled using Xenium (right) and segmented cells color coded by identity (left). Scale bars in (A) and (B) are 100 μm. (**C**) The density of eEVTs decreases in disease (not statistically significant). (**D**) Frequency of neighborhoods of eEVTs and endothelial cell types normalized by the total number of both of these cell types. (**E**) eEVTs in early PE have significantly down-regulated markers of cell adhesion in both spatial domains, decidua distal (top) and muscle (bottom). (**F**) eEVTs in early PE also down-regulate or up-regulate several cytokines (*P < 0.05, GLM). Expression is normalized.

Spiral artery remodeling also involves the replacement of maternal vasculature by endothelial EVTs (eEVTs) that accumulate around maternal arteries (*5*). In our single-cell reference, we identified a small cluster of these cells (*5*, *18*, *34*–*43*), but their low number (*n* = 38) precluded meaningful comparisons among cases and controls. To better quantify spatial differences of this important but rare cell type in disease, we again used in situ spatial transcriptomics at a single-cell resolution (Xenium). We identified eEVTs in the distal decidua (*n* = 314 in cases and *n* = 74 in controls) and muscle (*n* = 383 and *n* = 16, respectively) (fig. S20C), including around the maternal vasculature (Fig. 4, A and B).

In severe PE, eEVTs are sparse in both spatial domains in the myometrium (Fig. 4C), similarly to EVTs (Fig. 3D and fig. S20, D and E). However, to quantify their association with the maternal vasculature, we directly measured how often they are nearest neighbors with endothelial cells. As expected, these neighborhoods decrease as a function of distance to the villi. Still, they decrease more in severe PE (Fig. 4D), suggesting reduced association with maternal vasculature in early disease.

We also performed differential gene expression on eEVTs in the two spatial domains of the myometrium (table S7). We found that eEVTs down-regulate, in early disease, cell-to-cell adhesion molecules. This agrees with a previous report (*5*). Down-regulated genes include *Cadherin-5* (*CDH5*), *Intercellular Adhesion Molecule-1* (*ICAM1*), and *NCAM1* (*Neural Cell Adhesion Molecule 1*) (Fig. 4E). In addition, an important molecule implicated in extracellular matrix and spiral artery remodeling, *Matrix metalloproteinase-12* (*MMP12*) (*34*), decreased its expression significatively in early PE across spatial domains (Fig. 4E). The same trend has been observed in maternal circulation for PE (*35*).

Similarly, we detected overall down-regulation of cytokines and interleukins in early disease (Fig. 4F), important mediators of immune regulation through pregnancy (*36*, *37*). For example, in the decidua distal, we found down-regulation of the *C-C motif chemokine receptor1* (*CCR1*), which has been implicated in impaired trophoblast migration (*38*). We also detected down-regulation of *C-X-C motif chemokine ligand 12* (*CXCL12*), reported before in severe PE (*39*). *CXCL12* could mediate interactions of eEVTs and endothelial cells as suggested from ligand-receptor analysis (*5*).

In the myometrium, we detected elevated levels of the pro-inflammatory *C-X-C motif chemokine ligand 10* (*CXCL10* or *IP-10*) and *CXCL11* (or *I-TAC*) on eEVTs in disease (Fig. 4F), mirroring results from maternal circulation in severe PE (*40*). Similarly, there is up-regulation of the pro-inflammatory cytokine *Interleukin-6* (*IL6*), also found elevated in maternal serum in severe PE (*41*). *IL6* has been implicated in inflammation and vascular remodeling during pregnancy complications (*42*). However, there is also down-regulation in disease of the pro-inflammatory gene *Tumor Necrosis Factor Receptor Superfamily Member 1B* (*TNFRSF1B*) in both spatial domains of the myometrium, pointing out to the complex interplay between cytokines and PE (Fig. 4F and table S7) (*43*). Together, our analysis suggests that impaired cell adhesion and altered inflammation in eEVTs contribute to their low association to vascular cell types, likely affecting spiral artery remodeling in early PE.

### Extraplacental trophoblasts in the decidua parietalis are spatially patterned

**Fig. 5.**
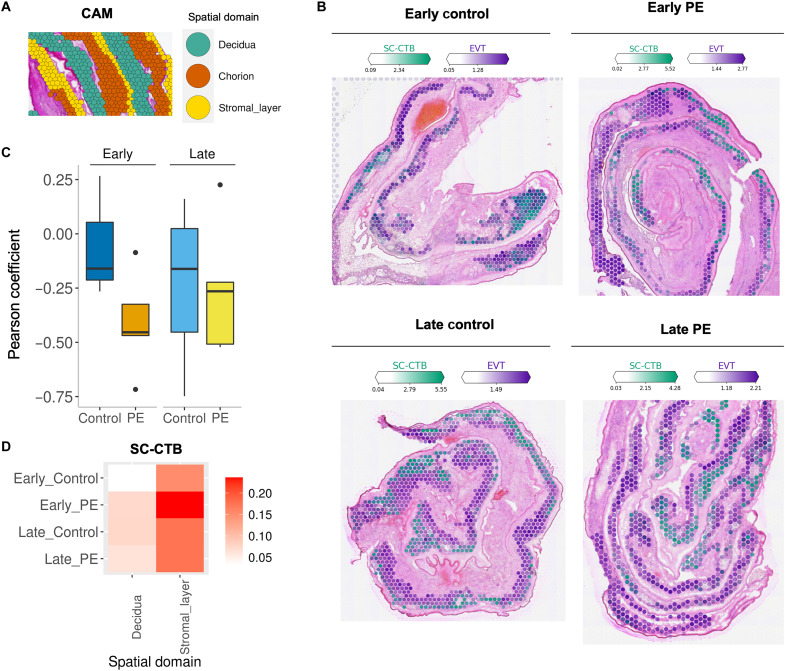
Extraplacental trophoblasts in the smooth chorion have a distinct spatial distribution. (**A**) Spatial domains at the CAM are the maternal decidua parietalis, the fetal chorion, and the fetal stromal layer (within a rolled CAM). (**B**) SC-CTBs and EVTs colored by their deconvoluted abundance in the chorion. (**C**) Pearson correlation coefficient of SC-CTBs and EVTs located in the chorion, which is negative, suggesting that they are spatially anticorrelated. (**D**) Neighboring spots of those with high SC-CTB abundance, expressed as a proportion. The SC-CTBs are closer to the stromal layer than the decidua.

The FMI also extends to the CAM, where we defined spatial domains of fetal and maternal origin (Fig. 5A). In the chorion (fetal), we identified EVTs that are transcriptionally similar to those at the decidua basalis, as previously reported (*44*). We also confirmed a recently described population of trophoblasts, the smooth chorion CTBs (SC-CTBs), characterized by the expression of keratins *KRT6A*, *KRT14*, and *KRT17* and the integrin subunit *ITGB6* (fig. S10A) (*44*). We noticed that the tissue distribution of EVTs and SC-CTBs is spatially anticorrelated (Fig. 5, B and C), particularly in early PE. This is the result of SC-CTBs in early disease favoring the stromal layer (Fig. 5D), stratifying their distribution in the CAM.

### Numerous maternal immune cell types have mitochondrial dysfunction

**Fig. 6.**
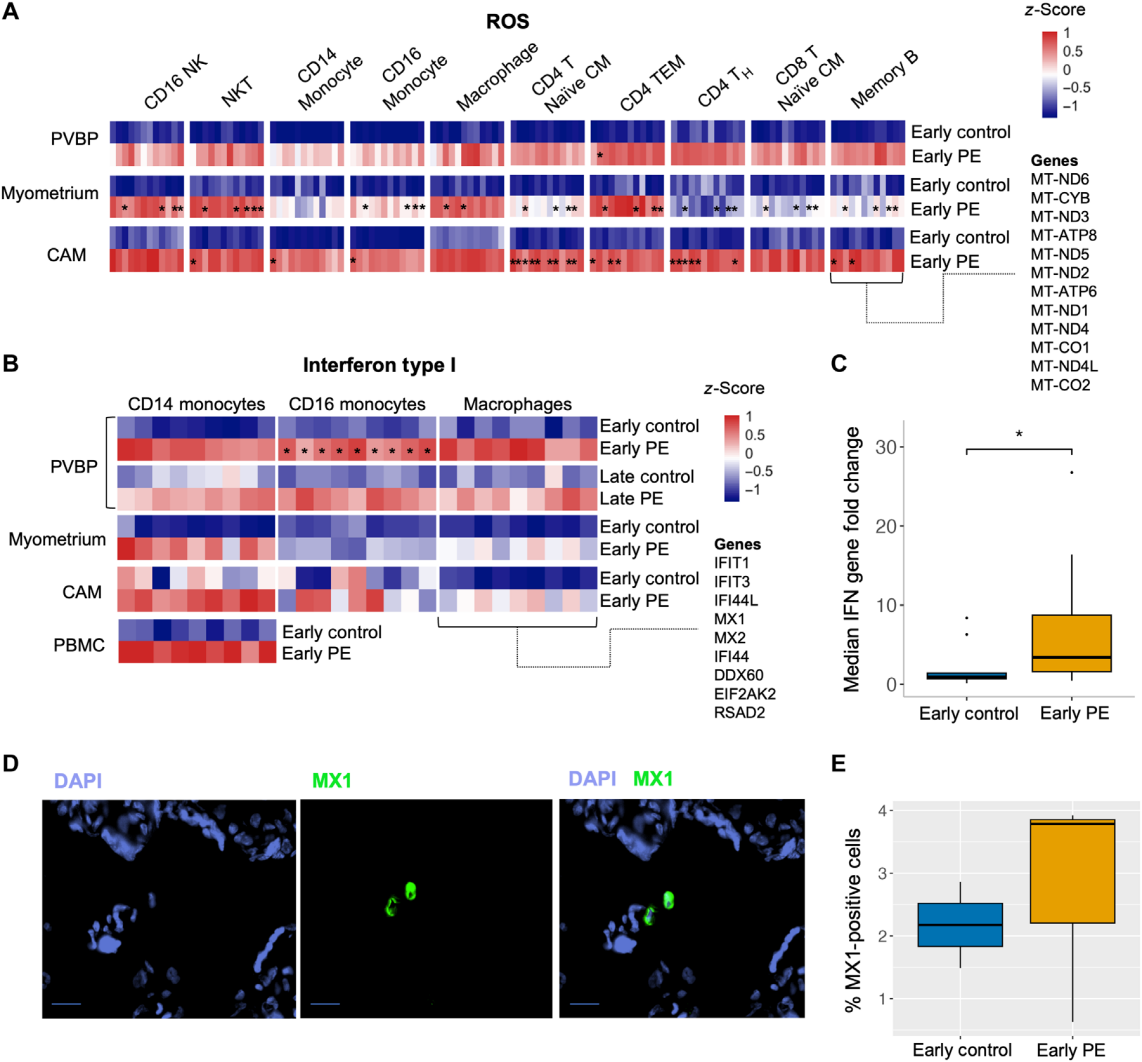
Maternal immune effects in severe PE across the FMI. (**A**) Mitochondrial genes involved in oxidative stress genes are up-regulated in multiple cell types in early disease. Gene expression is normalized and scaled using *z*-score across conditions per gene (*P < 0.05, GLM). (**B**) IFN-stimulated genes (ISGs) are up-regulated in several myeloid cell types. (**C**) qPCR of ISGs in PBMCs, with significant up-regulation in early disease (*P < 0.05, Wilcoxon rank sum). (**D**) Example of immunohistochemistry in the PVBP in early PE of an ISG (MX1, green) and DAPI (blue). Scale bars, 20 μm. (**E**) Immunofluorescence quantification of MX1-positive cells in the PVBP for two cases and three controls in early disease.

Inflammation and alterations in several maternal immune cell types have been reported in severe PE (*45*). We found, in early disease, up-regulation of genes involved in mitochondrial function and oxidative stress across maternal immune cell types in the PVBP, myometrium, and CAM (Fig. 6A and fig. S21A; *P* values in tables S5 and S6). These are genes involved in the production of reactive oxygen species (ROS) (several subunits of complex I nicotinamide adenine dinucleotide and hydrogen dehydrogenase: *MT-ND1*, *MT-ND2*, *MT-ND3*, *MT-ND4*, *MT-ND4L*, and *MT-ND6*), *cytochrome c oxidase subunit 1 and 2* (*MT-CO1* and *MT-CO2*), *cytochrome b* (*MT-CYB*), and *mitochondrial ATP synthase complex* (*MT-ATP6* and *MT-ATP8*). Affected cell types include both innate cell types (NK cells, NKT cells, and macrophages) and adaptative immune cell types (CD8 and CD4 T cells). Dendric cell types (pDC, cDC1, cDC2, and cDC3) are at low numbers in our analysis, yet they also present mitochondrial alterations (fig. S21A). This indicates broad immune maternal mitochondrial dysfunction across the FMI in early PE. This may also be the case in some fetal immune populations (fig. S21D).

### Type I IFN is up-regulated in the myeloid compartment

We also identified enrichment of IFN-stimulated genes (ISGs) in maternal CD14 monocytes, CD16 monocytes (*P* < 0.05), and macrophages in the PVBP in early and late disease (Fig. 6B and fig. S21B). The ligand-receptor activity of ISGs also supports an enhanced type I IFN (IFN-I) response in the myeloid compartment (fig. S21C). Moreover, MX1 immunofluorescence (representative ISG) supports up-regulation of IFN-I (Fig. 6, D and E). Notably, there is up-regulation of ISG in CD14 monocytes and also in the myometrium, CAM, and PBMCs, although it did not reach significance (Fig. 6B). Using an extended cohort, we thus corroborated it in maternal circulation by quantitative polymerase chain reaction (qPCR) in early disease (Fig. 6C and table S4). Thus, there is a pathological IFN-I response in the myeloid compartment across the FMI, one that is detectable in maternal circulation within 25 to 34 weeks of gestation. Notable is the complementary and significant up-regulation of *IFITM3* in fetal monocytes as well (fig. S21E).

### Macrophages at the PVBP up-regulate genes associated with cell death and fibrosis

**Fig. 7.**
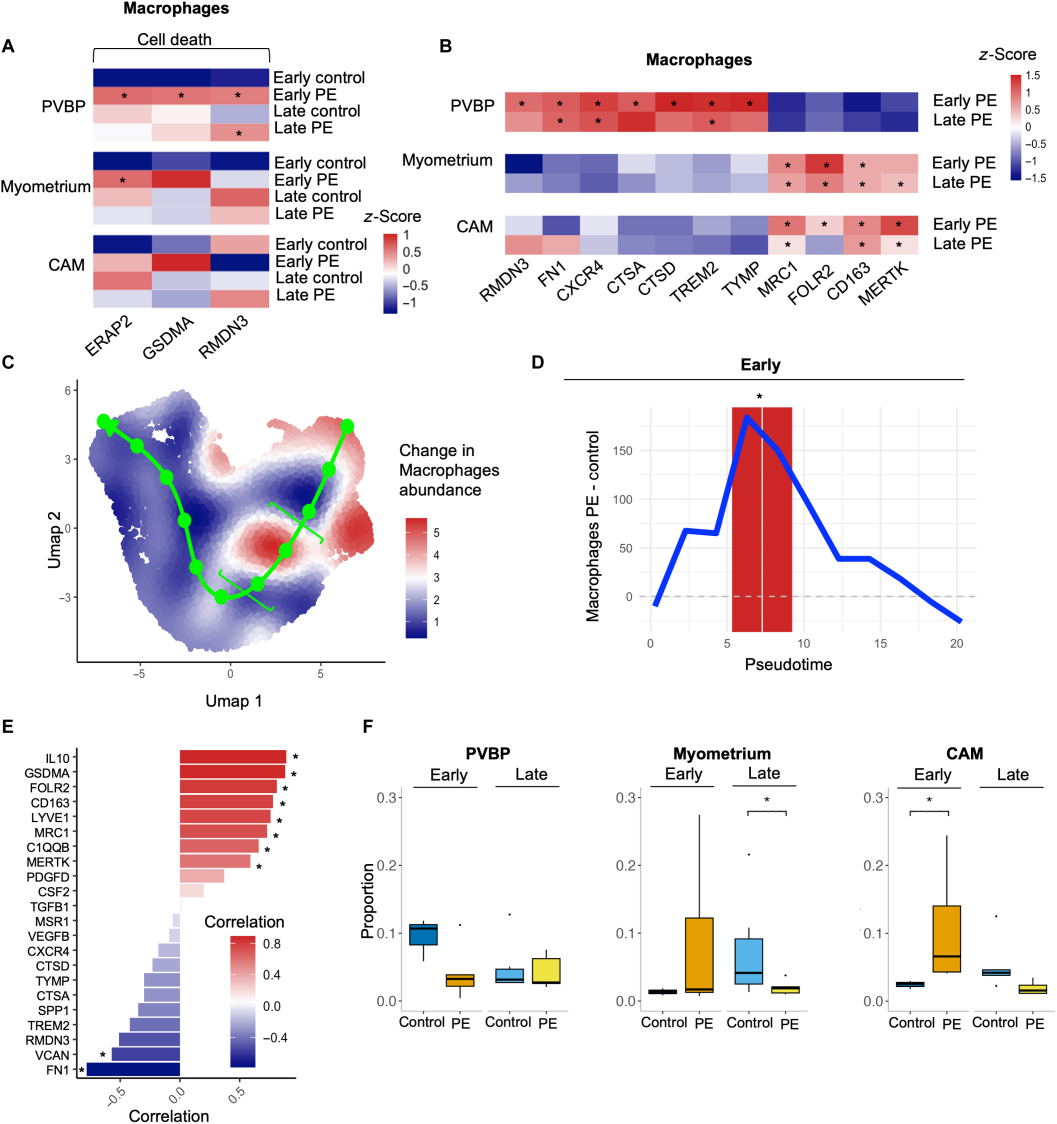
Maternal macrophage signatures in severe PE across the FMI. (**A**) Macrophages up-regulate genes related to cell death. (**B**) Macrophages in severe PE have gene expression differences across the FMI. Gene expression is normalized and scaled using *z*-score across tissues per gene (*P < 0.05). (**C**) UMAP of changes in macrophage abundance in PE against controls along the inferred macrophage pseudotime trajectory (green line). The pseudotime interval considered for downstream analysis is in brackets. (**D**) Difference of cell counts in early disease (blue line). The red background indicates pseudotime intervals where the cell count difference is significant (Wilcoxon rank-sum test, *P < 0.05). (**E**) Correlation between the gene expression and the cell abundance along pseudotime colored by significance from a Pearson correlation test. (**F**) Proportion of macrophage in early and late disease per tissue (*P < 0.05).

We then investigated maternal macrophage responses at the FMI. This highly heterogeneous cell type has been implicated in pregnancy complications, including PE (*7*, *46*). We identified, with our GLM, significant up-regulation of *Gasdermin A* (*GSDMA*) in early disease at the PVBP, with also elevated expression in the myometrium and CAMs (Fig. 7A). *GSDMA* is a pore-forming protein in the mitochondrial membrane involved in pyroptotic cell death (*47*). Two other genes whose overexpression leads to apoptosis, the mitochondrial *RMDN3* (*PTPIP51*) (*48*) and *Endoplasmic reticulum aminopeptidase 2* (*ERAP2*) (*49*), are also significantly up-regulated in early PE at the PVBP (Fig. 7A). In agreement, macrophages in this tissue are reduced (Fig. 7F), possibly from selective cell death.

Furthermore, the anti-inflammatory molecule *TREM2* expression is up-regulated in early (*7*) and late PE in the PVBP (Fig. 7B). Macrophages in the PVBP also overexpress several proteases and scavenger proteins, including cathepsins (*CTSD* and *CTSA*) and *MSR1* (CD204) (*50*), as well as profibrotic *SPP1*/*osteopontin*, *FN1*, and *CXCR4* (Fig. 7B) (*51*–*53*). This suggests that macrophages in the PVBP are affected and likely contribute to placental fibrosis, a cardinal feature of early PE.

### Macrophages at the myometrium and CAM up-regulate phagocytic and anti-inflammatory genes

Meanwhile, macrophages at the myometrium and CAM have similar gene expression (Fig. 7B), including significant up-regulation of phagocytosis gene *MRC1* (CD206) and anti-inflammation genes *CD163*, *FOLR2*, and *MERTK* (*51*, *54*, *55*) in early and late PE. There is also a subpopulation of macrophages in the myometrium and CAM (Fig. 7, C and D) up-regulating additional anti-inflammatory genes, such as *IL10* and *LYVE1* (Fig. 7E) (*54*, *55*). Toward this end, macrophages expand in early PE in the myometrium and CAM, the opposite behavior of macrophages in the PVBP (Fig. 7F). Together, these suggest that macrophages are distinctly activated across the FMI in disease. They decrease through apoptosis in the PVBP, while promoting fibrosis, and expand in the myometrium and CAM to fight inflammation. These are hallmarks of lost homeostasis in these tissues under severe PE.

### Long-range effects of trophoblasts in severe PE

**Fig. 8.**
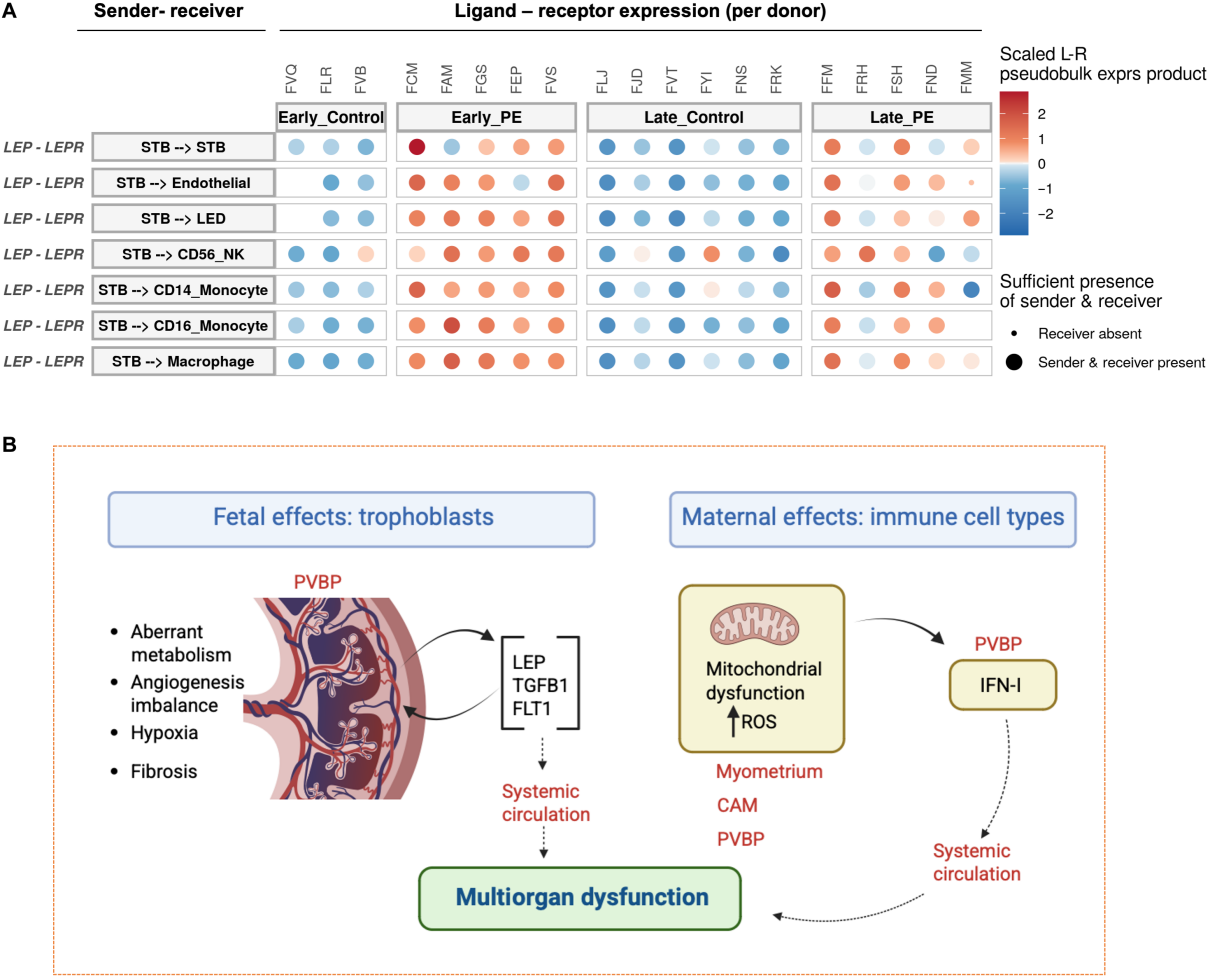
Trophoblast long-range effects might affect maternal cell types in severe PE. (**A**) Ligand-receptor communication, per donor, between STBs expressing *LEP* and immune cell types expressing its receptor *LEPR*. Interactions with a prioritization score above 0.5 are shown. The dot plot depicts scaled ligand-receptor pseudobulk expression product. (**B**) Fetal effects from the villous tree include the release of molecules to systemic circulation, affecting multiple organs. Among the maternal effects in immune cell types, we uncover mitochondrial dysfunction and IFN-I expression. We detect IFN-I in maternal circulation, further contributing to the systemic and multiorgan pathology of severe PE. Created in BioRender. S. Castellano (2026), https://BioRender.com/pqdj877.

Some of the products of the up-regulated genes we identified in STBs, for example, *LEP*, *IGF2*, and *FLT1* (Fig. 2), have been identified in maternal circulation (*3*, *21*), perhaps contributing to the systemic pathophysiology of severe PE. We thus assessed cell-to-cell communication from STBs to other cell types, jointly in the consecutive sections of the PVBP and myometrium. We identified the *LEP* receptor, *LEPR*, highly expressed in monocytes and macrophages as well as in endothelial and NK maternal cells in early and late PE. Notably, *LEPR* is also expressed in STBs, suggesting a positive feedback loop enhancing *LEP* effects (Fig. 8A). We also identified *TGFB1* receptors (produced by STBs) in some myeloid and endothelial cells (fig. S22). In the endothelial cells of the myometrium, we also detected down-regulation of genes associated with angiogenesis (fig. S21I). So, in addition to the well-known systemic endothelial dysfunction effect of *FLT1* (*3*), other long-rage effects from the villi to myeloid and endothelial cell types may contribute to the severe PE presentation.

## DISCUSSION

Sampling all the tissues from the FMI, while correcting for gestational age, allowed us to identify the distinct fetal and maternal molecular contributions to severe PE. We spatially and quantitatively characterized them, revealing broad dysregulation of the placental villi, where a combination of angiogenic, metabolic, fibrotic, and hypoxic fetal alterations accompany the disease (Fig. 8B). We confirmed the reduced invasiveness of EVTs in severe PE (*2*), which becomes more pronounced distally from the villous region. We also quantified molecular changes in eEVTs, an important cell type that directly contributes to the remodeling of the spiral arteries. Notably, eEVTs in the myometrium have impaired cell adhesion and an increased pro-inflammatory profile in severe PE, likely contributing to the inadequate remodeling of the maternal vasculature.

In addition, *LEP* is strongly up-regulated at the placental villi in early and late disease. This is important as leptin induced reactive oxygen species, unfolded protein response, and ER stress, as well as vascular endothelial dysfunction, in mice (*20*, *21*). In this context, our inferred *LEP* cell-to-cell communication within STBs may enhance placental dysfunction. In addition, *LEP* communication to endothelial and immune cell types in the adjacent decidua and myometrium, together with the reported leptin increases in maternal serum of PE (*21*), may cause systemic effects (Fig. 8B). Leptin antagonists have shown promise in autoimmune diseases (*56*) and are an exciting avenue for STB targeted therapies.

We reveal among the maternal contributions to disease a novel IFN-I response in the myeloid compartment, along with mitochondrial oxidative stress in numerous immune cell types in the myometrium, CAM, and PVBP in early disease (Fig. 8B). This is in addition to the known mitochondria dysfunction and oxidative stress in STBs (*57*, *58*). This maternal oxidative stress is implicated in the activation of IFN-I in several immune conditions with systemic inflammation, such as systemic lupus erythematous (*59*) and juvenile dermatomyositis (*60*). Notably, patients with systemic lupus erythematous are at an increased risk of developing PE and have up-regulated IFN signaling that contributes to vascular damage at the FMI (*61*). Elevated IFN-I also resulted in abnormal EVT migration in an in vitro system (*62*). We thus propose IFN-I as an unrecognized contributor to systemic endothelial dysfunction in severe PE. Its presence in peripheral blood in severe PE, also demonstrated here, opens exciting future research on the quantification of IFN-I peripherally over the pregnancy. IFN-I in peripheral blood promises better screening of patients that might benefit from anti-IFN (*63*) or antioxidant therapeutics (*60*, *64*).

Further to ectopic IFN-I in the diseased myeloid compartment, we also unraveled maternal responses in macrophages across tissues at the disease FMI. At the PVBP, macrophages express profibrotic molecules that may contribute to the placental fibrosis observed in histology. The hypoxic tissue environment and IFN/mitochondrial stress can trigger selective apoptosis (*65*), decreasing the response to local inflammation despite the expression of anti-inflammatory molecules such as *TREM2* (*7*). In contrast, myometrial and CAM macrophages express a molecular signature, including *MERTK*, *MRC*, and *LYVE*, that in rheumatoid arthritis potentially resolves inflammation and contributes to remission (*55*). As new cell type–specific treatments emerge, we advance that some macrophage subpopulations may be a therapeutic target (*66*, *67*).

While our single-cell analysis allowed for well-powered comparisons across the FMI, to which we added spatial context, this work has some limitations. Some cell types may not be well represented, with cells above a certain size or with complex shapes more likely to be excluded (*66*, *67*). In addition, despite the identification of meaningful molecular signatures in eEVTs using newer spatial technologies at a single-cell resolution, the number of donors in this analysis is too small for identifying nuanced gene expression differences. Future application of spatial technologies at a single-cell and subcellular resolution in larger cohorts may reveal novel signatures of disease.

In conclusion, we present novel mechanistic insights into severe PE, a disease orphan of treatment, opening different tissue- and cell-specific responses for therapeutic consideration in the future. Given the severity of molecular dysfunctions in early disease, compared to its late presentation, timely intervention during gestation is likely beneficial and could change the extremely poor prognosis of severe PE.

## MATERIALS AND METHODS

### Experimental design: Donor inclusion and exclusion criteria

We recruited pregnant women at the fetal medicine unit, antenatal care unit, and labor ward of the University College London Hospitals, NHS Foundation Trust. The study was approved by the South Oxford Research Ethics Committee (REC CODE: 17/SC/0432). PE is diagnosed as a new onset of maternal hypertension (*3*, *68*). Severe PE is defined as PE with a blood pressure of 140/90 mmHg and end-organ dysfunction or biochemical abnormalities or a blood pressure of 160/110 with proteinuria and/or other severe features (see Table 1) (*14*, *69*). The study cohort comprised 20 pregnant women, 10 of which were diagnosed with severe PE and 10 gestational matching controls ranging from 25 to 37 weeks of gestational age. Donors were divided into early (≤34 weeks) and late (>34 weeks) disease or controls on the basis of gestational age at delivery. We used gestational age at delivery of cases, instead of disease onset, to be able to match them within 1 week of gestational age with their respective controls, minimizing the confounding effect of gestational age, which we show to be profound (Table 1 and Supplementary Text).

Inclusion criteria were women with a singleton pregnancy, able to consent, experiencing preterm delivery by C-section because of iatrogenic reasons or severe PE before 37 weeks of gestation. Exclusion criteria were women with pregnancies affected by major fetal anomalies (whether chromosomal, structural, or genetic), twin pregnancies and comorbidities including diabetes mellitus (gestational or T1 or T2), and maternal autoimmune, renal, or heart disease. Key inclusion as a control was recruitment where placental pathology was not thought to be driving disease (no placental pathology identified from history or ultrasound) and no clinical signs of growth restriction. Donors were excluded if they had any clinical symptoms/signs of infection or a positive test of infection during standard clinical practice. In addition, we ruled out possible undetected infections using metagenomics. Our metagenomics analysis identified *Ureaplasma* pathogenic bacteria in donor FJJ, which was then excluded from our comparative analysis but included in the FMI atlas.

### Tissue sampling

PVBP samples were collected from the midpoint of the largest distance between the cord insertion site and the edge of the placenta. Myometrial samples were taken via biopsy from the area of the placental bed mirroring the expected placental sampling site. From the edge of the placenta closest to the placental tissue sampling site, CAMs were cut from the placental edge and rolled, with the end closest to the placenta being in the middle of the roll. For each tissue, adjacent samples were collected, one was placed in cold HypoThermosol FRS for dissociation and single-cell transcriptomics, and another was snap frozen and embedded in optimal cutting temperature medium for spatial transcriptomics. Up to 24 hours before delivery, 7 ml of peripheral venous maternal blood was collected for PBMC isolation. Further details are provided in Supplementary Text.

### Power analysis

To calculate the power of detecting differences on our cohort on a hypothetical common cell type (10%) or a rare cell type (2.5%), we simulated fold changes and sample sizes using the R package scPower_1.0.1 (*70*). Despite some biological simplifications, on the basis of the number of donors and under optimal sequencing depth, we calculated the power of detection from single-cell transcriptomics for comparisons of 8 (early disease) and 11 samples (late disease). Assuming a significance threshold of 0.05, considering “expressed genes” as genes with more than three counts in more than 50% of the total samples, the power to detect a gene with a difference of a 2.8-fold change in common cell type (10% of the population) is 0.86 (8 samples) and 0.90 (11 samples), while the detection power of a rare cell type (2.5% of the population) is 0.41 (8 samples) and 0.58 (11 samples). Further details are provided in Supplementary Text.

Smaller differences in rare cell types are theoretically more challenging to detect using 8 to 11 samples. Increasing the sample size would solve this problem. For instance, the power to detect a difference of 1.5 log fold change in a rare cell type (2.5%) increases with 400 individuals from 0.04 and 0.06 (8 and 11 samples, respectively) to 0.81 (400 samples). However, recruiting this number of patients and processing their samples would have made this research impractical. Thus, we argue that the sample size used has detection power for early and late disease and balances it with recruitment time and cost.

### Metagenomics

PVBP and CAM samples for each donor were processed including an additional patient (FCK) that had clinical signs of infection and acted as a positive control. Parallel DNA and RNA extractions using the ZymoBIOMICS DNA/RNA Miniprep Kit were performed. RNA and DNA libraries were prepared as previously described for Illumina sequencing (*71*) with microbial enrichment using the NEBNext Microbiome DNA Enrichment Kit following the manufacturer’s protocol but using double the amount of MBD2-Fc–bound magnetic beads per nanogram. Enriched DNA was processed with the NEBNext Ultra II FS DNA Library Prep Kit for Illumina. RNA libraries were prepared using the KAPA RNA HyperPrep Kit with RiboErase. RNA and DNA libraries from each batch were sequenced either on the Illumina Nextseq 2000 (pilot and batch 1) or Illumina Novaseq 6000 (batches 2 and 3) to achieve between 20 million and 50 million reads per sample (table S1). These were demultiplexed and ran through the CZID web-based platform for taxonomic profiling (*72*) and filtering using the package Metathresholds (*71*).

### Single-cell tissue dissociation

PVBP, CAM, and myometrial samples were dissociated on a gentleMACS Octo dissociator in 10 ml of Accutase (PVBP and CAM) or 5 ml of Accumax (myometrium) using a custom protocol (see Supplementary Text). Dissociated cell suspensions were then passed through stacked 100- and 70-μm cell strainers and subjected to red cell lysis using Red Cell Lysis buffer (Miltenyi Biotec). PVBP and CAM samples were then subjected to dead cell removal using Dead Cell Removal MicroBeads loaded onto MACS LS columns (Miltenyi Biotec). Myometrial samples were pelleted and resuspended in 1× Dulbecco’s phosphate-buffered saline (DPBS) and 0.04% bovine serum albumin and passed through a 40-μm Flowmi Cell Strainer (Sigma-Aldrich). In addition**,** nucleus single-cell suspensions were prepared for the CAM from donors “FVQ,” “FCM,” “FGC,” “FLJ,” “FRK,” and “FRH”, as single-cell libraries proved challenging. Briefly, snap-frozen and optimal cutting temperature–embedded CAM samples were cryosectioned, and nuclei were isolated from the sections using the Chromium Nuclei Isolation Kit (10x Genomics), following 10x Genomics user guide CG000505. PBMCs from peripheral venous maternal blood were isolated using Histopaque 1077 (Sigma-Aldrich). Single-nucleus samples span across early and late PE and controls, introducing no bias to their comparison.

### Single-cell and spatial transcriptomic library preparation and sequencing

Dissociated cells, isolated PBMCs, and isolated nuclei were checked for concentration and viability using an Acridine Orange/Propidium Iodide Stain (Logos Biosystems) on a Logos Biosystems Luna FL automated cell counter. Single-cell suspensions per tissue and donor were used to generate single-cell/nucleus transcriptomics. We loaded 20,000 cells/nuclei into a 10x Genomics Chromium Controller using the Chromium Next GEM Chip K and Chromium Next GEM Single Cell 5′ Kit v2 kits (10x Genomics) as per 10x Genomics user guide CG000331.

Ten-micrometer sections of snap-frozen PVBP, myometrium, and CAM samples were loaded onto Visium Spatial Gene Expression slides as recommended by 10x Genomics. The area of the PVBP samples chosen for loading onto the relevant capture areas contained the edge proximal to the decidua basalis. Loaded sections on Visium slides were fixed, stained, and imaged on a Motic EasyScan One slide scanner. Spatial transcriptomic libraries were prepared following the manufacturer’s guidelines. Permeabilization times of 18 min for CAM and myometrium and 6 min for PVBP, were established as appropriate using the Visium Spatial Tissue Optimisation kit (10x Genomics).

Resulting spatial, single-cell, and single-nucleus transcriptomics were sequenced on an Illumina Novaseq 6000 using five S4 (200 cycles) version 1.5 sequencing kits (Illumina), with a configuration of read 1: 28 cycles; index read 1: 10 cycles; index read 2: 10 cycles; and read 2: 190 cycles. Libraries were sequenced to a minimum coverage of 20,000 reads per cell or 25,000 reads per Visium spot.

### In situ spatial transcriptomics assay

Ten-micrometer sections of the tissue myometrium from two early controls (donors “FVB” and “FVQ”) and four early PE (donors “FCM,” “FEP,” “FAM,” and “FVS”) were loaded onto specialized slides to be processed on the 10x Genomics Xenium Analyzer. We used the Xenium Human Immuno-Oncology panel and a Xenium Add-on Custom 100 Gene Panel, following 10x Genomics handbook CG000579 and user guides CG000749 and CG000584.

### Immunofluorescence assay

PVBP samples from early PE and early controls were cryosectioned and loaded onto one glass slide. Sections were fixed in 10% formaldehyde and then blocked and permeabilized using 1× DPBS, 0.1% Tween, 10% fetal bovine serum, and 0.1% Triton-X with dextran sulfate (10 mg/ml) for 60 min. Overnight staining was performed using 1× DPBS, 0.1% Tween, and 10% fetal bovine serum with dextran sulfate (10 mg/ml) and a 1:100 dilution of CoraLite Plus 488–conjugated MX1 Recombinant antibody (Proteintech). Then, nuclear stain 4′,6-diamidino-2-phenylindole (DAPI) was added at 5 μg/ml for 1 min, and a coverslip was then added using SlowFade Gold Antifade mounting medium. Stained slides were imaged on a Nikon Ti2 inverted microscope at 20× magnification. Resulting image files were loaded into QuPath 0.5.0. Morphological regions were delineated to include an approximately equivalent proportions of decidua and villous placenta to capture both maternal and fetal contributions. Positive cell detection analysis was performed using DAPI for nucleus detection and a nuclear maximum intensity threshold for detection of MX1-positive cells.

### Quantification of IFN-I expression in PBMCs

An extended cohort of early PE (*n* = 14) and gestational matched controls (*n* = 9) was used to measure IFN-I expression in PBMCs (see details in Supplementary Text and table S4). PBMCs isolated from whole blood were subjected to RNA extraction using the Qiagen RNEasy Plus kit. Extracts were then subjected to qPCR using taqman probes for four IFN-related genes (*IFIT1*, *IFI44L*, *ISG15*, and *RSAD2*) along with one housekeeping gene (18*S*). The NEB Luna Universal Probe One-Step RT-qPCR Kit was used, and qPCR reactions were performed using an Applied Biosystems Quantstudio 5 Real-Time PCR instrument, using cycling conditions as per recommended for the reaction kit. Output files were processed using Design & Analysis Software by Applied Biosystems with Ct (cycle threshold) values per replicate group exported as a .csv file. DDCt values were calculated and then converted into fold change values as per 2^DDCT^. The median value of the above four IFN-related genes per sample was calculated. The Wilcoxon test was used to compare cases and controls for each gene, along with the median IFN gene fold change.

### Alignment, count matrix, and ambient correction of single-cell RNA/single-nucleus RNA sequencing data

For each single-cell and single-nucleus library, the raw bcl files were converted into fastq files using mkfastq (version cellranger-7.1.0; 10x Genomics). Sequencing reads were aligned to the GRCh38-2020-A human reference genome (GENCODE version 32/Ensembl98 distributed by 10x Genomics), and a matrix of unique molecular identifier counts per library was obtained using cellranger “multi” (table S2). We used CellBender 0.2.0 to correct for ambient RNA per library and classify cell-containing droplets from empty ones (*73*).

### Fetal and maternal cell classification in single-cell transcriptomics

For cell classification into fetal or maternal, we used Freemuxlet version 1 (popscle software) (*15*). First, a variant site list (VCF) with the most common single-nucleotide polymorphisms (SNPs) from the 1000 Genomes Project was used as a panel of SNP positions. Then, the dsc-pileup function was used to identify the allelic composition, base quality, and the number of reads from each allele at each common SNP location per library using the BAM file output from cellranger. We combined the dsc-pileup outputs from the tissue PVBP with myometrium, PBMCs, or CAM per patient to circumvent the low abundance or absence of fetal cells in the myometrium and PBMCs and run Freemuxlet using two groups (i.e., two individuals: --nsample 2) and default parameters.

To identify the inferred genotype (0, 1, or 0/1) that corresponded to the fetus or mother or doublet, we calculated the average expression of a set of trophoblast markers (“*CGA*,” “*CYP19A1*,” “*GH2*,” “*PAPPA*,” “*VGLL1*,” “*PAPPA2*,” and “*HLA-G*”) per genotype. We assigned as fetal the genotype with higher average trophoblast expression. To verify the Freemuxlet genotype assignment, we used a DNA microarray (Infinium Global Diversity Array version1.0) on a subset of samples (eight pairs of fetus and maternal donors using DNA isolated from cord blood and PBMCs, respectively).

### Single-cell quality control

Doublets were detected in two ways: (i) using Scrublet 0.2.3 (manual threshold = 0.25) (*74*) per library and (ii) as a droplet with a mixed genotype (0/1) from Freemuxlet. These were filtered out. Seurat 4.2.1 (*75*) was used to calculate quality control metrics. Cells were filtered out if (i) the number of detected genes was below 400 and above 6000, (ii) the number of unique molecular identifiers was above 30,000, (iii) the percentage of mitochondrial reads was more than 12%, and (iv) the percentage of hemoglobin genes was more than 0.25.

### Single-cell integration and cell type annotation

After preprocessing, gene expression matrices from all donors and all tissues were integrated using Seurat 4.2.1 (*75*). Briefly, a standard workflow including normalization (“LogNormalise”), scale (“ScaleData”), and highly variable gene selection (2000 genes) was used to perform principal components analysis (npcs = 25), *k*-nearest neighbor calculation (k.param = 20), and graph-based community detection using Louvain clustering. Harmony 0.1.0 (*76*) was used to correct for batch effects from the single-cell gene expression and single-nucleus gene expression method differences. No other variables showed a batch effect.

We annotated cell types using gene expression of manually curated genes from the literature and genes differentially expressed per cluster (obtained using the Seurat function “FindMarkers”). Cell types were annotated at three levels of resolution (refereed as annotation levels 1, 2, and 3). First, a coarse cell group annotation rendered six major groups: (i) trophoblasts, (ii) stromal, (iii) vascular, (iv) myeloid, (v) B, and (vi) T/NK cells at annotation level 1. Subsetting each of these groups and repeating the clustering pipeline described above allowed the assignment of two more granular levels of annotation. During clustering, we detected further doublets (identified by a mixed profile of gene expression in the cluster), and those were also filtered out.

### Cell abundance differences from disease and gestational age

To measure the differential abundance of cell populations at a single-cell resolution because of disease or gestational age, we used Dawnn 1.0.8 (*77*). Using as an input a Seurat object per tissue, we calculated a *k*-nearest neighbor graph using the first 10 principal components (reduced_dim = pca) for the PVBP, myometrium, and PBMC tissues. For the tissue CAM, we used Harmony 0.1.0 (reduced_dim = harmony) (*76*) to correct for batch effects introduced by the two different single-cell methods and used the first 10 components from Harmony. We applied a false discovery rate correction with the Benjamini-Yekutieli procedure as implemented in Dawnn 1.0.8 using an alpha = 0.05.

We tested for differential abundance separately comparing (i) conditions (control or PE) or (ii) gestational age (early or late). We measured whether a cell is called differentially abundant in one comparison, in both, or in neither. Donor FJJ was excluded of this and the rest of the comparative analysis, as explained above.

### Cell type differential gene expression analysis per tissue

We tested for single-cell differential gene expression in each tissue while accounting for gestational effects and donor-to-donor variability. Cell types with less than 25 cells per condition or present in only one donor were excluded from the differential expression analysis. Genes lowly expressed (less than 20 total counts) were also filtered out.

For each tissue, we first aggregated the measured counts per donor and cell type at annotation level 3 into a pseudobulk expression profile. We use EdgeR to fit a log-linear model using the design matrix: ∼0 + *c*, where *c* corresponds to the condition

$$c=\left[\text{Earl}y_{\text{control}},\;\text{Earl}y_{\text{PE}},\;\text{Lat}e_{\text{control}},\;\text{Lat}e_{\text{PE}}\right]$$

We defined early disease as the contrast Early_PE_ vs Early_control_ and late disease as Late_PE_ vs Late_control_.

We tested for differential expression per contrast using a likelihood ratio test implemented in EdgeR 3.36.0 (*78*). Multiple testing was controlled by using a Benjamini-Hochberg correction. Genes were considered differentially expressed if the log fold change >0.5 and false discovery rate <0.05 (tables S5 and S6).

### Macrophage differential expression across tissues

To test for differences in gene expression of macrophages across tissues, we also used a GLM framework (EdgeR). Selecting only PE macrophages (early PE or late PE), we modeled gene expression using a paired design to adjust for donor-specific effects using the design matrix ∼ 0 + tissue + donor. As before, we used a log-linear model for tissues *k* = 3, and the contrasts were set to compare macrophages from PVBP vs myometrium and PVBP vs CAM.

### Cell proportions per tissue

To test for differences in cell proportions accounting for donor variability and gestational age, we used the GLM framework implemented in the propeller function of the R package speckle 0.0.3 (*79*). We use a matrix with donors *d* as rows and conditions *c* as columns to fit a linear model using the design matrix ∼ 0 + *c*.

For tissue = {CAM}, we added a term to correct for the effect introduced by using two different single-cell methods using the modified design matrix ∼ 0 + *c* + method. We tested for differences in cell proportions per cell type using a moderate *t*-statistical test per contrast and applied a false discovery rate correction using a Benjamini-Hochberg procedure.

### Differential abundance and gene expression in pseudotime in STBs and macrophages

We used slingshot version 2.6.0 (*80*) to infer cell trajectories in pseudotime following the Condiments pipeline (*81*) to identify pseudotime intervals (as a proxy of cell subpopulations) containing differential abundance of cells of any condition (control or severe PE). We used in-house R scripts to generate a sequence of pseudotime intervals of *n* pseudotime units (by dividing the maximum pseudotime by 10 to obtain *n* intervals). For each interval, we obtained the number of cells from each donor or the proportion of cells relative to the donor’s total number of cells. To test for difference in cell counts (or proportions) in early or late disease, we used a Wilcoxon test followed by a Benjamini-Hochberg correction for multiple testing (threshold, 0.2) performed in intervals with cells from at least three donors from each condition.

To correlate shifts in gene expression with shifts in cell abundance across pseudotime intervals, we calculated the mean gene expression of a given gene (or set of genes) per cell at each pseudotime interval and correlated this expression with the difference between the mean of cells per donor in severe PE cases compared to controls in each interval.

### Cell-to-cell communication

MultiNicheNet version 1.0.3 (*82*) was used for differential ligand-receptor analysis in early and late disease. Cell types from the tissues PVBP and myometrium were integrated into a single Seurat object to take advantage of their spatial continuity. Cell types at annotation level 3 with less than 25 cells and coming from one single donor were excluded. The Nichenet version 2 ligand-receptor network and matrices from “organism = human” were obtained from Zenodo (DOI: 10.5281/zenodo.7074291). Early disease and late disease were modeled using the same GLM as before. We applied a *P* value cutoff of 0.05 and a log fold change threshold of 0.5. Other parameters were set as default. We selected the top 150 targets per ligand and visualize the top predictions using MultiNicheNet in-built functions.

### Alignment and gene count per spot of Visium

Demultiplexing, alignment of reads to the human reference genome (GRCh38-2020-A, GENCODE version 32/Ensembl98 distributed by 10x Genomics), identification of spots for each tissue, and quantification of gene counts per spot were performed using spaceranger version 2.0.0 (10x Genomics).

### Spatial domain identification per tissue

Spatial domains were identified manually by two researchers using the 10x Genomics Loupe Browser 7. Annotations were cross-referenced to generate a consensus. First, a *k*-means clustering of the gene expression per capture area (*k* = 4 for CAM and *k* = 3 for PVBP and myometrium tissues) was used to generate clusters that broadly aligned with the morphologically expected tissue domains. A combination of differential expression analysis (in-built in Loupe browser) and knowledge of cell type markers with known spatial distribution (i.e., decidual cells in the decidua basalis and decidua parietalis from PVBP and CAM, respectively) was used to manually annotate spatial domains. Poor quality spots, artifacts, or low-confidence spatial domain assignment was manually labeled as “background” and was excluded from further analysis.

### Spatial hypoxia gene expression score in placenta villi

The hypoxia score included the genes “*HTRA1*,” “*HTRA4*,” “*FSTL3*,” “*EGLN3*,” and “*TMEM454*” and was calculated using the Seurat function AddModuleScore (*75*) using 30 control genes on spots belonging to the spatial domain “villi” per capture area.

### Cell type deconvolution

cell2location version 0.1.3 (*83*) was used to deconvolute cell type abundances per spot in Visium. We trained a single-cell regression model for 350 epochs using the cell type annotation at level 2 as a single-cell reference (excluding donor FJJ), keeping genes expressed in at least 3% of the cells and excluding genes expressed in less than five cells. To deconvolute the cell types per spot, we use N_cells_per_location = 8 and detection_alpha = 20. We trained the deconvolution model for 3000 epochs and exported the 5% quantile of posterior distribution for visualization. Each Visium capture area was deconvoluted separately.

### Spatial expression statistics using a mixed-effects model

To test for differences in abundance of EVT per spot in early or late disease, we used a linear mixed-effects model implemented in the packages lme4 1.1-27.1 and lmerTest 3.1-3. We considered the condition (early control versus early PE or late control versus late PE) as the fixed effect, while the variation between donors belonging to the same condition was considered a random effect using the model

$$\begin{array}{c} \text{lmer}\left(\left(c2\text{loc\_EVT}\right)∼\text{condition}+\left(1|\text{donor}\right)\right) \end{array}$$

### Spatial correlation of EVTs and SC-CTBs in the CAM

A Pearson correlation was used to quantify spatial correlation of EVT and SC-CTB in the CAM spatial domain “chorion.” We filtered out spots with SC-CTB abundance less than 0.5. For a statistical comparison between conditions in early or late disease, we used Fisher’s *Z*-transformation and computed a *P* value (two-tailed test).

### Neighborhood analysis in the CAM

To quantify the spatial arrangement of spots with high abundance of the cell type SC-CTB (abundance greater than 3) in the spatial domain “chorion” (CAM), we calculated the identity of their neighbor spots using the nearest-neighbor algorithm (*k* = 2), as implemented in the RANN 2.6.1 package.

### Single-cell analysis of in situ spatial transcriptomics

Cell type annotation of Xenium datasets was performed using a standard clustering pipeline, as described above for single-cell transcriptomics. Spatial domain identification was done manually using Xenium Explorer 4. The cell density was calculated as the number of cells divided by the area of the spatial domain. Quantification of cell neighborhoods between eEVTs and endothelial cells used the nearest-neighbor algorithm (*k* = 2), as implemented in the RANN 2.6.1 package. The number of nearest neighbors was expressed as a proportion of the total eEVTs and endothelial cells sampled. Differential expression analysis was performed using the same GLM applied to the single-cell analysis.

## Supplementary Materials

### The PDF file includes:

Supplementary Text Figs. S1 to S22 Table S1 Legends for tables S2 to S8 References
